# A Breakdown Enhanced AlGaN/GaN Schottky Barrier Diode with the T-Anode Position Deep into the Bottom Buffer Layer

**DOI:** 10.3390/mi10020091

**Published:** 2019-01-26

**Authors:** Youlei Sun, Ying Wang, Jianxiang Tang, Wenju Wang, Yifei Huang, Xiaofei Kuang

**Affiliations:** The Key Laboratory of RF Circuits and Systems, Ministry of Education, Hangzhou Dianzi University, Hangzhou 310018, China; 161040013@hdu.edu.cn (Y.S.); hztangjx@hdu.edu.cn (J.T.); 162040172@hdu.edu.cn (W.W.); 162040160@hdu.edu.cn (Y.H.); kuangxiaofei@hdu.edu.cn (X.K.)

**Keywords:** T-anode, GaN, buffer layer, anode field plate (AFP), cathode field plate (CFP)

## Abstract

In this paper, an AlGaN/GaN Schottky barrier diode (SBD) with the T-anode located deep into the bottom buffer layer in combination with field plates (TAI-BBF FPs SBD) is proposed. The electrical characteristics of the proposed structure and the conventional AlGaN/GaN SBD with gated edge termination (GET SBD) were simulated and compared using a Technology Computer Aided Design (TCAD) tool. The results proved that the breakdown voltage (V_BK_) in the proposed structure was tremendously improved when compared to the GET SBD. This enhancement is attributed to the suppression of the anode tunneling current by the T-anode and the redistribution of the electric field in the anode–cathode region induced by the field plates (FPs). Moreover, the T-anode had a negligible effect on the two-dimensional electron gas (2DEG) in the channel layer, so there is no deterioration in the forward characteristics. After being optimized, the proposed structure exhibited a low turn-on voltage (V_T_) of 0.53 V and a specific on-resistance (R_ON,sp_) of 0.32 mΩ·cm^2^, which was similar to the GET SBD. Meanwhile, the TAI-BBF FP SBD with an anode-cathode spacing of 5 μm achieved a V_BK_ of 1252 V, which was enhanced almost six times compared to the GET SBD with a V_BK_ of 213 V.

## 1. Introduction

At present, most power semiconductor devices are fabricated from Si materials, but as the process progresses, the performance of Si devices is approaching the material limit. Therefore, wide-bandgap semiconductor materials such as diamond [1,2], SiC [3,4], and GaN [5] have become promising candidates to make high power semiconductor devices. These wide-bandgap semiconductor materials have a high breakdown field [2], high thermal conductivity [6,7], and an extremely low intrinsic carrier concentration at room temperature, which can make power devices with high potential figures of merit [2]. However, GaN power devices such as AlGaN/GaN HEMT and Schottky barrier diodes (SBD) have attracted more attention, as the GaN heterojunction can make them have a faster switching speed, high breakdown voltage, and low on-resistance [8,9,10,11,12,13]. In this work, an AlGaN/GaN SBD will be investigated in detail. Despite the advantage mentioned above, there are still many unsolved problems before AlGaN/GaN SBD can be used on a large scale, such as the high turn-on voltage, high anode leakage, and low breakdown voltage. By now, numerous approaches like the etching barrier layer [13] and selective Si diffusion [14] have been demonstrated to effectively reduce the onset voltage, but the leakage current is relatively large. In addition, combinations of high/low Schottky barrier metals [15], carbon-doped GaN buffer [16,17], Fe-doped GaN buffer [18], and gated edge termination (GET) [19,20] have been proposed to suppress the reverse leakage, but the double Schottky barrier metal and GET are still a challenging manufacturing issue, and the doped GaN buffer will affect the device’s forward characteristics, especially the on-resistance. The utilization of field plates (FPs), such as anode FPs (AFP) [21] and cathode FPs (CFP), is a simple and effective method to modulate the electric field. In our previous work, vertical FPs (VFP) [22] were proposed for the redistribution of the electric field in the anode–cathode region. However, the effect of a vertical FP is much lower than a lateral FP, and the VFP does not contribute to suppressing the anode leakage current.

In this work, a T-anode located deep into the bottom buffer layer of the SBD in combination with field plates (TAI-BBF FPs SBD) is proposed where the T-anode not only plays the role of VFP, but also introduces the leakage electrons into the bottom buffer layer with high concentration acceptor traps, resulting in the significant suppression of the anode leakage current. As a result, the 2DEG in the channel can be depleted and the space charge region will be extended, which leads to the enhancement of the breakdown voltage. Compared to the previous work, double GaN buffers were selected for the T-anode located deep into the bottom buffer layer of the SBD (TAI-BBF SBD). The first buffer layer with low concentration acceptor traps is called the middle buffer layer, and the second buffer layer with high concentration acceptor traps is called the bottom buffer layer, and can not only suppress the leakage current, but also has a negligible effect on the device’s forward characteristics. Moreover, the combination of the T-anode and the bottom buffer layer can cause most of the leakage electrons to be trapped in the bottom buffer. Meanwhile, the electrons in the bottom buffer layer can effectively modulate the device’s surface electric field. In addition, the GET is integrated into the Schottky contact (SC) serving as the anode [19,20] to reduce the turn-on voltage. The AFP along with the CFP are located separately at the anode and cathode, which can modulate the electric field distribution in the anode–cathode region, which in turn results in an appreciable V_BK_ improvement. More importantly, the proposed structure has a minor influence on the forward characteristics, thus obtaining a huge breakdown voltage while maintaining a low R_ON,sp_.

## 2. Device Structure and Simulation Model

**Figure 1:**
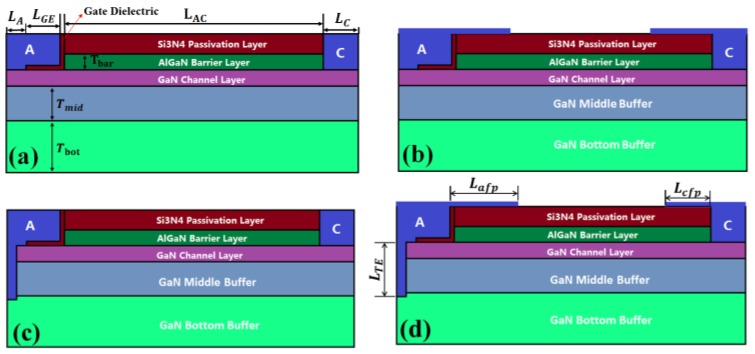
Cross-section of: (**a**) Schottky barrier diode (SBD) with gated edge termination (GET SBD), (**b**) SBD with gated edge termination in combination with field plates (GET FPs SBD), (**c**) T-anode located deep into the bottom buffer layer of the SBD (TAI-BBF SBD), (**d**) T-anode located deep into the bottom buffer layer of the SBD in combination with field plates (TAI-BBF FP SBD).

Devices with identical physical dimensions consisting of a conventional GET SBD [20], a SBD with gated edge termination in combination with field plates (GET FPs SBD), TAI-BBF SBD, and a TAI-BBF FPs SBD are shown in Figure 1.

These architectures in the simulation included a passivation layer, an unintentionally-doped AlGaN barrier layer/GaN channel layer, and a buffer layer. Si_3_N_4_ of the size 100 nm was selected as the passivation layer [23] and the AlGaN contained a 0.25 Al mole part. In addition, the dielectric layer in the GET was also Si_3_N_4_ and the thickness was set to 20 nm. The passivation layer and Al_0.25_Ga_0.75_N barrier layer under the anode were etched completely in order to achieve a low V_T_. The proposed device had an additional T-anode with an initial length of 0.445 μm connecting the Schottky contact with the bottom buffer and two field plates located at the anode and cathode. The buffer layer was divided into a middle buffer and a bottom buffer [17]. The donor impurity would inevitably be introduced in the process of device growth, so the donor concentration in all GaN layers was set to 1 × 10^16^ cm^−3^ [17] to emulate the donor impurity. In order to reduce the buffer layer leakage, acceptor type traps were doped into both the middle buffer layer and the bottom buffer layer. The effective concentration of the acceptor type traps of 2 × 10^16^ cm^−3^ [10] and 4 × 10^18^ cm^−3^ [16,17] were selected to dope into the middle and bottom buffer layers, respectively, in order to neutralize the dopants and reduce the buffer layer leakage. Therefore, the middle and bottom buffer layers became slight p-type and heavy p-type layers, and the density of the hole provided by the two buffer layers could reach 2 × 10^16^ and 4 × 10^18^ cm^−3^ [16,24], respectively. The middle buffer close to the channel layer could suppress the leakage current effectively, but it had an imperceptible impact on the 2DEG because of the slight p-type. In contrast, the heavy p-type bottom buffer layer combining with the T-anode could play a leading role in reducing the anode leakage current. Meanwhile, the bottom buffer layer had little effect on the 2DEG, due to its distance from the channel layer. In addition, the energy level of the acceptor trap was set at E_V_ + 0.9 eV [17,25,26] and the cross-section σ_n_ was selected at 1.3 × 10^−14^ cm^−2^ [17] in this simulation, because in the previous report [25,26], the energy level of the acceptor trap was defined at E_V_ + 0.86 eV to E_V_ + 0.93 eV. Other device parameters along with their values used in the simulation are listed in Table 1.

The Sentaurus software was selected for 2D numerical simulation. Some necessary physics models [27,28] were adopted, such as mobility models, the Shockley–Read–Hall recombination model, and a polarization model. Furthermore, in the AlGaN/GaN SBD, tunneling leakage at the Schottky junction played the leading role in the reverse leakage current [29], so a nonlocal tunneling model [27] was set at the Schottky contact, and the work function of Schottky anode was defined to be 4.6 eV [20].

### Fabrication Process

**Figure 2:**
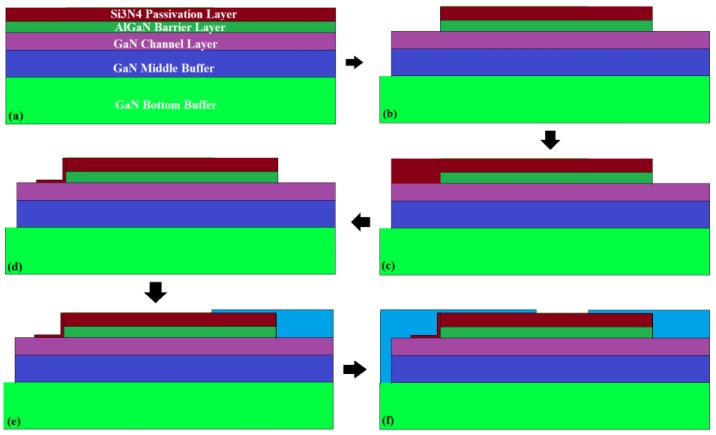
Schematic of the fabrication process steps for the proposed structure.

In order to explain how the proposed device could be implemented, a brief schematic of the fabrication process steps is presented in Figure 2.

The corresponding description of these process steps is summarized as follows.

(a) Metal organic chemical vapor deposition (MOCVD) was adopted to grow the base structure including the GaN bottom buffer layer, the GaN middle buffer layer, the GaN channel layer, and the AlGaN barrier layer, and the Si_3_N_4_ passivation layer was then deposited using plasma-enhanced chemical vapor deposition (PECVD) [30].
(b) The Si_3_N_4_ passivation layer, AlGaN barrier layer, GaN channel layer, and GaN buffer layer were etched through inductively coupled plasma reactive ion etching (ICP RIE), using a BCl_3_/Cl_2_ gas mixture [30].
(c) The Si_3_N_4_ layer was deposited on the anode region using plasma-enhanced chemical vapor deposition (PECVD) [30].
(d) ICP RIE was adopted to etch the Si_3_N_4_ layer of the anode region and kept at a thickness of 20 nm for the Si_3_N_4_ layer at the right and bottom side.
(e) A Ti/Al/Ni/Au ohmic metal was deposited using e-beam evaporation on the cathode, followed by rapid thermal annealing at 800 °C for 30 s in N_2_ ambient [12]. Lastly, the Schottky metal stack of Ni/Au (40 nm/350 nm) was deposited [12].

The above fabrication process was relatively easy to implement, the only difficulty was that the depth of the groove was not well controlled when etching the T-anode groove. Fortunately, when the T-anode reached 0.445 µm, the breakdown voltage almost reached the saturation value, so there was a certain fault tolerance to the depth of the etched groove.

## 3. Results and Discussion

### 3.1. Forward and Reverse Characteristics

**Figure 3:**
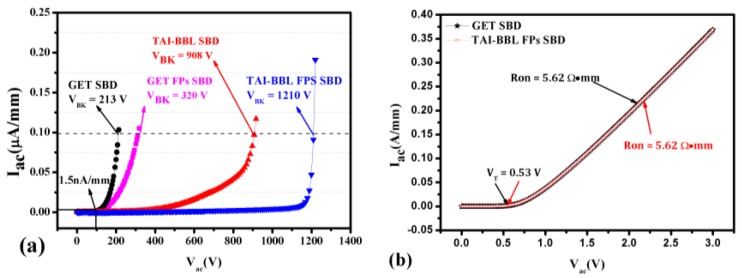
(**a**) The breakdown characteristics of the GET SBD, GET FPs SBD, TAI-BBF SBD and TAI-BBL FPs SBD; (**b**) The forward characteristics of the GET SBD and TAI-BBL FPs SBD.

The breakdown and forward characteristics of the GET SBD, GET FPs SBD, TAI-BBF SBD, and TAI-BBF FPs SBD are shown in Figure 3.

The breakdown criterion of all the devices was when the anode leakage current reached 0.1 μA/mm, as shown in Figure 3a. In contrast to GET SBD, with a V_BK_ of 213 V, the V_BK_ was enhanced to 320 V in the GET FP SBD, implying that the improvement of V_BK_ was inconspicuous. However, the V_BK_ was improved to 908 V in the TAI-BBF SBD and further promoted to 1210 V in TAI-BBF FP SBD. In addition, the leakage current reached 1.5 nA/mm when the reverse voltage was 100 V, which was consistent with the experimental results in [20]. The voltage of the forward current reaching 1 mA/mm was defined as the V_T_. As shown in Figure 3b, the V_T_ and R_on_ of the proposed structure were 0.53 V and 5.62 Ω·mm, similar in magnitude to the GET SBD, indicating that the proposed structure showed significant improvement in the reverse characteristics while maintaining the forward characteristics.

### 3.2. Equipotential Line and Horizontal Electric Field Distribution

**Figure 4:**
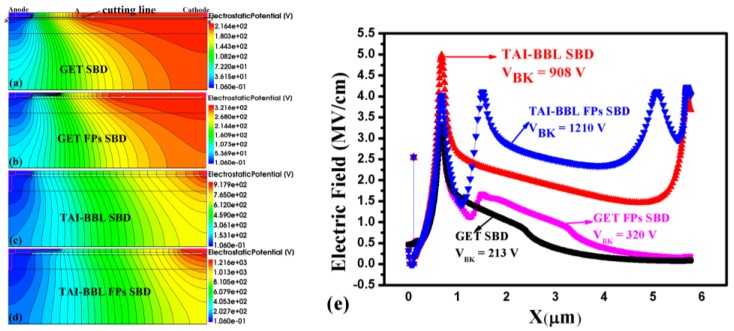
The equipotential line profile of: (**a**) GET SBD, (**b**) GET FP SBD, (**c**) TAI-BBF SBD, (**d**) TAI-BBF FP SBD when breakdown occurs, (**e**) The corresponding horizontal electric field distribution in the 2DEG area.

The equipotential line and horizontal electric field distribution of four devices when the breakdown occurred are described in Figure 4 to account for the enhancement of the breakdown characteristics. In the GET SBD, the equipotential lines from the anode to point A were very compact. They became sparse after point A, indicating that there were still a large number of electrons in the channel and there was little space charge created from point A to the cathode while the breakdown took place. In the GET FP SBD, the AFP and CFP were added to the GET SBD to modulate the electric field focused on the right of anode and the left of the cathode. However, the V_BK_ of the GET FP SBD only achieved minor improvement. As the electrons in the channel were not being depleted, the FPs did not work out as desired. In contrast to the GET SBD, the introduction of a T-anode in the TAI-BBF SBD resulted in the direct connection of the anode to the bottom buffer layer. A bottom buffer layer with a substantial amount of acceptor traps could help deplete the 2DEG in the channel to expand the space charge region, which could make the equipotential lines more compact throughout the anode–cathode region. Furthermore, the AFP and CFP were placed on the anode and cathode separately in order to make the equipotential line more uniform and denser throughout the whole TAI-BBF FP SBD, as shown in Figure 4d. As a result, the V_BK_ in the TAI-BBL FP SBD was promoted further compared to the GET SBD and TAI-BBL SBD.

A horizontal cutting line aa’ at 1 nm below the channel of the three devices was carried out to obtain the horizontal electric field distribution, as depicted in Figure 4e. In the GET SBD, the electric field peak occurred at the GET and the field declined sharply to a very small value on the right. In the GET FP SBD, a new field peak occurred to the right of the GET, but the value of the field peak was very small and it then dropped to a small value. In the TAI-BBF SBD, there was another electric field peak at the cathode because almost all of the 2DEG in the channel was depleted by the bottom buffer layer via T-anode, which resulted in the flattening of the electric field contribution in the anode–cathode region, consequently improving V_BK_. However, the electric field peak at the anode was too high, which resulted in the GET being punctured in advance. In the TAI-BBF FP SBD, the electric field at the GET declined and a new electric field peak appeared below the AFP and CFP due to electric field modulation. After the optimization described above, the V_BK_ became further enhanced as the electric field distributed more uniformly over the anode–cathode area.

### 3.3. Electron Concentration Distribution

**Figure 5:**
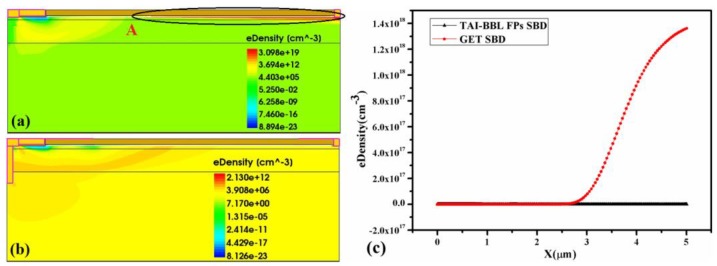
The electron concentration distribution of: (**a**) GET SBD, (**b**) the TAI-BBL FP SBD when the breakdown occurs, (**c**) the corresponding curve distribution.

The electron concentration distribution of the GET SBD and TAI-BBL FP SBD when the breakdown occurred are depicted in Figure 5 to explain the role of the T-anode. In the GET SBD, the electron in the channel from the anode to point A was depleted, but from point A to the cathode it was not, as can be seen at the black circle. In addition, there was a very low electron density in the buffer layers, especially the bottom buffer layer, which meant that the high concentration of traps in the buffer layers did not capture the leakage electron as desired. Therefore, there was no space charge generated from point A to the cathode to bear the breakdown voltage, due to the heavy leakage current when the breakdown occurred. In contrast, in the TAI-BBL FP SBD, the electron in the channel was fully depleted, as can be seen in Figure 5c. Moreover, the buffer layers were full of the leakage electron, which implied that the high concentration traps in the buffer layers captured the leakage electron effectively with the assistance of the T-anode. Consequently, the space charge region was extended from point A to the cathode in the TAI-BBL FP SBD resulting in the breakdown voltage being enhanced.

### 3.4. The Path of the Leakage Current and the Vertical Electric Field Distribution

**Figure 6:**
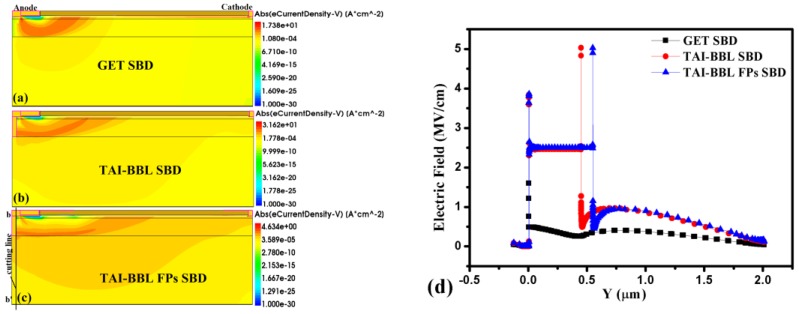
The path of the anode leakage current of: (**a**) GET SBD, (**b**) TAI-BBF SBD, (**c**) TAI-BBF FP SBD, (**d**) the corresponding vertical electric field distribution under the SC.

The path of the anode leakage current due to the tunneling process [20] and the vertical electric field distribution below the SC during breakdown were generated in order to explain the function of the T-anode directly, as shown in Figure 6.

In the GET SBD, the leakage current tunneled from the SC through the channel and middle buffer layer, and then to the cathode. Thus, the bottom buffer layer had little effect on suppressing the leakage current. In contrast, a part of the leakage current still tunneled through the channel and middle buffer layer to the cathode, but most of the leakage current tunneled from the terminal of the T-anode through the bottom buffer layer to the cathode in the TAI-BBL SBD and the TAI-BBL FP SBD. Therefore, the leakage was significantly suppressed and the V_BK_ was enhanced. A cutting line bb’ of the vertical electric field distribution for all devices was made in the middle of the SC during the breakdown, as shown in Figure 6c. Compare to the GET SBD, the value of the electric field in the vertical orientation was much larger in the TAI-BBF SBD and TAI-BBF FP SBD, which corroborates that the T-anode played the role of a vertical plate [22], so that the electrons were more able to tunnel from the termination of the T-anode into bottom buffer layer. As a result, the anode leakage current was suppressed and the V_BK_ was enhanced. However, if the T-anode only reached the channel layer or the middle buffer layer, the bottom buffer layer would not work as desired and the V_BK_ would decrease, as most of the electrons would tunnel from the T-anode through into the channel and middle buffer layer. Hence, the concentration of electrons in the middle buffer of the TAI-BBL SBD and TAI-BBL FP SBD were much higher than that of the GET SBD, which resulted in the device being punched through in advance.

## 4. Parameter Optimization

**Figure 7:**
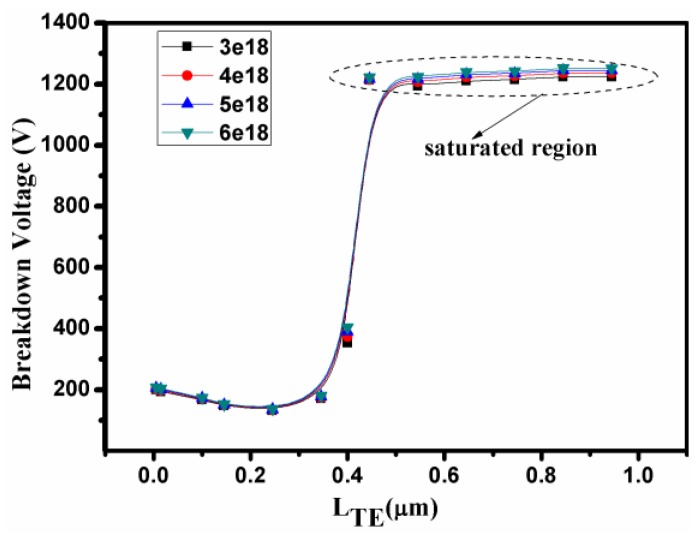
Dependence of the breakdown characteristics on the length of the T anode and the concentration of the acceptor traps of the bottom buffer layer.

The dependence of the breakdown characteristic on the length of the T-anode and the concentration of the acceptor traps in the bottom buffer layer are plotted in Figure 7. Initially, the length of the T-anode from 0 to 0.3 μm was far from the bottom buffer layer, resulting in a low V_BK_. When the length of the T-anode exceeded 0.3 μm, the V_BK_ began to increase sharply. Eventually, the V_BK_ achieved a saturation value when the length of T-anode reached 0.845 μm. Thus, the V_BK_ depended on the distance between the T-anode and the bottom buffer layer, as is evident from Figure 6. The concentration of acceptor traps versus the V_BK_ are also shown in Figure 7. However, when the concentration of the acceptor traps of bottom buffer layer increased from 3 × 10^18^ cm^−3^ to 6 × 10^18^ cm^−3^, the V_BK_ remained constant. With the doping concentration of the acceptor traps of 6 × 10^18^ cm^−3^, V_BK_ = 1252V was obtained for a T-anode length of 0.845 μm. In particular, changing the concentration of the acceptor traps of the bottom buffer layer had a negligible effect on the forward characteristics, as the bottom buffer layer was far from the GaN channel layer.

## 5. Conclusions

The function of the T-anode and the bottom buffer was discussed comprehensively in this work. The simulation results showed that the bottom buffer layer with high concentration acceptor traps was able to suppress the anode leakage current effectively via the T-anode, and the T-anode along with the AFP and CFP made the electric field contribution more uniform all over the anode–cathode region. The forward and breakdown characteristics of the GET SBD and the proposed structure were simulated and compared, demonstrating that the proposed structure was able to withstand a larger breakdown voltage while maintaining similar forward characteristics. Finally, a TAI-BBL FP SBD with L_AC_ = 5 μm achieved a V_BK_ of 1252V and a R_ON,sp_ of 0.32 mΩ·cm^2^, corresponds to the V_BK_ of 213V and R_ON,sp_ of 0.32 mΩ·cm^2^ of the GET SBD. This implies that a satisfactory trade-off between R_ON,sp_ and V_BK_ was obtained in the proposed structure.

## Figures and Tables

**Table 1:** Major optimized parameters of the proposed structure.

Parameter	Value
Anode length (L_A_)	0.2 μm
Gated edge length (L_GE_)	0.45 μm
Cathode length (L_C_)	0.1 μm
Anode–cathode spacing (L_AC_)	5 μm
T-anode length (L_T_)	0.445–0.945 μm
Barrier layer thickness (T_bar_)	25 nm
Channel layer thickness (T_chan_)	50 nm
Bottom buffer layer thickness (T_bot_)	1.6 μm
Middle buffer layer thickness (T_mid_)	0.4 μm
Cathode field plate length (L_cfp_)	0.6 μm
Anode field plate length (L_afp_)	0.83 μm

## References

[1] Holmes J., Dutta M., Koeck F.A., Benipal M., Brown J., Fox B., Hathwar R., Johnson H., Malakoutian M., Saremi M. (2018). A 4.5-μm PIN diamond diode for detecting slow neutrons. Nucl. Instrum. Methods Phys. Res. Sect. A.

[2] Saremi M., Hathwar R., Dutta M., Koeck F.A.M. (2017). Analysis of the reverse I-V characteristics of diamond-based PIN diodes. Appl. Phys. Lett..

[3] Mahabadi S.E.J., Moghadam H.A. (2015). Comprehensive study of a 4H–SiC MES–MOSFET. Phys. E.

[4] Moghadam H., Dimitrijev S., Han J., Han J., Haasmann D., Aminbeidokhti A. (2015). Transient-current method for measurement of active near-interface oxide traps in 4H-SiC MOS capacitors and MOSFETs. IEEE Trans. Electron Devices.

[5] Shen X.Q., Matsuhata H., Okumura H. (2005). Reduction of the threading dislocation density in GaN films grown on vicinal sapphire (0001) substrates. Appl. Phys. Lett..

[6] Anthony T.R., Banholzer W.F., Fleischer J.F., Wei L., Kuo P.K., Thomas R.L., Pryor R.W. (1990). Thermal diffusivity of isotopically enriched ^12^C diamond. Phys. Rev. B.

[7] Liu J., Ohsato H., Wang X., Liao M., Koide Y. (2016). Design and fabrication of high-performance diamond triple-gate field-effect transistors. Sci. Rep..

[8] Min W.H., Seung C.L., Young H.C., Soo S.K., Min K.H. (2006). New GaN Schottky barrier diode employing a trench on AlGaN/GaN heterostructure. Superlattices Microstruct..

[9] Zhu M., Song B., Qi M., Hu Z.Y., Nomoto K., Yan X.D., Cao Y., Johnson W., Kohn E., Jena D. (2015). 1.9-kV AlGaN/GaN Lateral Schottky Barrier Diodes on Silicon. IEEE Electron Device Lett..

[10] Jie H., Steve S., Ming Z., Andrea N.T., Isabella R., Matteo M., Xuan W.K., Benoit B., Denis M., Ben K. (2017). Time-dependent breakdown mechanisms and reliability improvement in edge terminated AlGaN/GaN Schottky diodes under HTRB tests. IEEE Electron Device Lett..

[11] Jun M., Elison M. (2017). High-voltage and low-leakage AlGaN/GaN tri-anode Schottky diodes with integrated tri-gate transistors. IEEE Electron Device Lett..

[12] Chuan W.T., Kai P.W., Yi W.L., Shawn S.H.H. (2016). 2.07-kV AlGaN/GaN Schottky barrier diodes on silicon with high Baliga’s figure-of-merit. IEEE Electron Device Lett..

[13] Treidel E.B., Oliver H., Rimma Z., Andreas W., Chafik M., Joachim W., Günther T. (2012). Fast-Switching GaN-Based Lateral Power Schottky Barrier Diodes With Low Onset Voltage and Strong Reverse Blocking. IEEE Electron Device Lett..

[14] Yi W.L., Yu S.L., Jui M.Y., Chih H.C., Shawn S.H.H. (2013). AlGaN/GaN Schottky Barrier Diodes on Silicon Substrates With Selective Si Diffusion for Low Onset Voltage and High Reverse Blocking. IEEE Electron Device Lett..

[15] Chang T.F., Huang C.F., Yang T.Y., Chiu C.W., Huang T.Y., Lee K.Y., Zhao F. (2015). Low turn-on voltage dual metal AlGaN/GaN Schottky barrier diode. Solid-State Electron..

[16] Eldad B.T., Frank B., Oliver H., Eunjung C., Joachim W., Günther T. (2010). AlGaN/GaN/GaN:C Back-Barrier HFETs With Breakdown Voltage of Over 1 kV and Low R_ON_ × A. IEEE Trans. Electron Devices.

[17] Xin L., Ying W., Fei C., Cheng H.Y., Xin X.F. (2017). A breakdown enhanced AlGaN/GaN MISFET with source-connected P-buried layer. Superlattices Microstruct..

[18] Chiu H.C., Chen S.C., Chiu J.W., Li B.H., Wang H.Y., Peng L.Y., Wang H.C., Hsueh K.P. (2018). AlGaN/GaN Schottky barrier diodes on silicon substrates with various Fe doping concentrations in the buffer layers. Microelectron. Reliab..

[19] Jing N.G., Mao J.W., Rui Y.Y., Shao F.L., Cheng P.W., Jin Y.W., Wen G.W., Yi L.H., Yu F.J., Bo S. (2017). Schottky-MOS Hybrid Anode AlGaN/GaN Lateral Field-Effect Rectifier With Low Onset Voltage and Improved Breakdown Voltage. IEEE Trans. Electron Devices.

[20] Jie H., Steve S., Silvia L., Brice D.J., Nicolò R., Andrea N.T., Dirk W., Shu Z.Y., Benoit B., Guido G. (2016). Statistical Analysis of the Impact of Anode Recess on the Electrical Characteristics of AlGaN/GaN Schottky Diodes With Gated Edge Termination. IEEE Trans. Electron Devices.

[21] Tang C., Xie G., Zhang L., Guo Q., Wang T., Sheng K. (2013). Electric field modulation technique for high-voltage AlGaN/GaN Schottky barrier diodes. Chin. Phys. B.

[22] Ying W., Zhi Y.L., Yue H., Xin L., Jun P.F., Ya C.M., Cheng H.Y., Fei C. (2018). Evaluation by Simulation of AlGaN/GaN Schottky Barrier Diode (SBD) With Anode-Via Vertical Field Plate Structure. IEEE Trans. Electron Devices.

[23] Hua M., Zhang Z., Wei J., Lei J., Tang G., Fu K., Cai Y., Zhang B., Chen K.J. Integration of LPCVD-SiNx gate dielectric with recessed-gate E-mode GaN MIS-FETs: Toward high performance, high stability and long TDDB lifetime. Proceedings of the IEEE International Electron Devices Meeting (IEDM).

[24] Kentaro H., Hiroshi O., Hirohumi T., Tohru N., Tomoyoshi M. Junction-Barrier Schottky Diodes Fabricated with Very Thin Highly Mg-Doped p + -GaN(20 nm)/n-GaN Layers Grown on GaN Substrates. Proceedings of the IEEE International Meeting for Future of Electron Devices, Kansai (IMFEDK).

[25] Honda U., Yamada Y., Tokuda Y., Shiojima K. (2011). Deep levels in n-GaN doped with carbon studied by deep level and minority carrier transient spectroscopies. Jpn. J. Appl. Phys..

[26] Lyons J.L., Janotti A., Walle C. (2010). Carbon impurities and the yellow luminescence in GaN. Appl. Phys. Lett..

[27] (2013). Sentaurus Device User Guide Version I-2013.12.

[28] Stephan S., Axel E., Tommaso C., Denis M., Steve S., Benoit B. TCAD methodology for simulation of GaN-HEMT power devices. Proceedings of the IEEE 26th International Symposium on Power Semiconductor Devices IC’s (ISPSD).

[29] Hu J., Lenci S., Stoffels S., Jaeger B.D., Groeseneken G., Decoutere S. (2014). Leakage-current reduction and improved on-state performance of Au-free AlGaN/GaN-on-Si Schottky diode by embedding the edge terminations in the anode region. Phys. Status Solidi C.

[30] Hyun S.L., Dong Y.J., Youngrak P., Jeho N., Hyun G.J., Hyoung S.L., Chi H.J., Junbo P., Sang O.R., Sang C.K. (2015). 0.34 V_T_ AlGaN/GaN-on-Si Large Schottky Barrier Diode With Recessed Dual Anode Metal. IEEE Electron Device Lett..

